# Finding Ideal Parameters for Recycled Material Fused Particle Fabrication-Based 3D Printing Using an Open Source Software Implementation of Particle Swarm Optimization

**DOI:** 10.1089/3dp.2022.0012

**Published:** 2023-12-11

**Authors:** Shane Oberloier, Nicholas G. Whisman, Joshua M. Pearce

**Affiliations:** ^1^ Department of Electrical and Computer Engineering, Michigan Technological University, Houghton, Michigan, USA.; ^2^ Department of Electrical and Computer Engineering, Ivey School of Business, Western University, London, Canada.

**Keywords:** particle swarm optimization, LDPE, waste plastic, recycling, fused particle fabrication

## Abstract

As additive manufacturing rapidly expands the number of materials including waste plastics and composites, there is an urgent need to reduce the experimental time needed to identify optimized printing parameters for novel materials. Computational intelligence (CI) in general and particle swarm optimization (PSO) algorithms in particular have been shown to accelerate finding optimal printing parameters. Unfortunately, the implementation of CI has been prohibitively complex for noncomputer scientists. To overcome these limitations, this article develops, tests, and validates PSO Experimenter, an easy-to-use open-source platform based around the PSO algorithm and applies it to optimizing recycled materials. Specifically, PSO Experimenter is used to find optimal printing parameters for a relatively unexplored potential distributed recycling and additive manufacturing (DRAM) material that is widely available: low-density polyethylene (LDPE). LDPE has been used to make filament, but in this study for the first time it was used in the open source fused particle fabrication/fused granular fabrication system. PSO Experimenter successfully identified functional printing parameters for this challenging-to-print waste plastic. The results indicate that PSO Experimenter can provide 97% reduction in research time for 3D printing parameter optimization. It is concluded that the PSO Experimenter is a user-friendly and effective free software for finding ideal parameters for the burgeoning challenge of DRAM as well as a wide range of other fields and processes.

## Introduction

The field of 3D printing is constantly expanding into new materials, such as biofilms,^1^ elastomer resins,^2^ dental materials,^3^ preceramic polymers,^4^ ceramics,^5^ silicone,^6^ and even edible inks.^7^ In addition, there are many conventional recycled thermoplastics,^8^ which are just now being embraced by the 3D printing community. These include not only the two most popular fused filament materials: polylactic acid (PLA)^9–12^ and acrylonitrile butadiene styrene,^13–17^ but also common thermoplastics such as high-density polyethylene (HDPE),^18–21^ polypropylene (PP) and polystyrene (PS),^20^ thermoplastic polyurethane,^22^ polyethylene terephthalate (PET),^23,24^ and polycarbonate (PC).^25^

The creation of recycled plastic filament extruders such as the open source recyclebots,^13,18,26^ which upcycle postconsumer plastic waste into 3D printing filament, also allows for the further democratization of distributed recycling and additive manufacturing (DRAM).^13,27,28^ Consumers have a direct economic incentive to recycle with DRAM^13,28^ because they can use their waste to fabricate many consumer products for far less than they can be purchased for from conventional manufacturing.^29–32^ DRAM thus has the potential to radically impact global value chains.^33^ In addition to reducing 3D printing costs by several orders of magnitude, DRAM decreases embodied energy of 3D printing filament by 90% thus radically improving the environmental impact.^34–36^

Unfortunately, each melt and extrude cycle of a recyclebot impairs the mechanical properties of PLA,^10^ HDPE,^21^ and even of PET.^23^ This limits the recycling cycles to approximately five^10^ before reinforcement or blending with virgin materials becomes necessary. Polymer composites using carbon-reinforced plastic,^37^ fiber-filled composites,^38,39^ and various types of waste wood^40,41^ have been used in recyclebot systems, and more complex DRAM systems can use 3D-printed PC as molds for intrusion molding^25^ for windshield wiper composites^42^ as well as acrylonitrile styrene acrylate and stamp sand waste composites.^43^ Zander *et al.*^44^ have studied PET, PP, and PS blends with styrene ethylene butylene styrene and maleic anhydride compatibilizers that were able to increase tensile strength.

DRAM presents a significant challenge to 3D printing operators. Postconsumer waste has a wide variety of contaminants, exact polymer specifications are unknown (even to manufacturers of 3D printing filament for 3D printed waste), the polymer history (e.g., number of cycles) may be unknown, etc.^45^

Many novel materials require unique end effectors,^46^ and there has been a rise of direct extrusion fused particle fabrication/fused granule fabrication (FPF/FGF)-based systems.^25,47–49^ A conventional method for finding idealized printing parameters is to print a consistent model using a matrix of parameters, effectively sweeping through every possible permutation.^47,50^ This is time consuming and inefficient, however and does not scale well across the myriad numbers of potentially recyclable waste plastic streams.

Computational intelligence (CI) can be leveraged to minimize the time it takes to optimize for process parameters experimentally.^51^ CI methods such as hierarchical machine learning,^6^ symbiotic organism search algorithms, and particle swarm optimization (PSO)^52^ have been proven as effective methods to find optimal printing parameters. In this context—a particle refers to a “candidate solution” that must be tested and will evolve over each iteration.

Typically, the implementation of the CI algorithm is prohibitively complex for noncomputer scientists, or in some cases the implementation of the algorithms is not open source. In this article, “PSO Experimenter,”^53^ an easy-to-use open-source platform based around the PSO algorithm, is introduced. Though PSO Experimenter is made for general implementations, the application of 3D printing is explored specifically.

In this study, PSO Experimenter is evaluated for the first time by applying it to known test functions, which have similar responses to 3D printing parameter sweeps. Then, PSO Experimenter is used to find optimal printing parameters for a relatively unexplored potential DRAM material that is widely available: low-density polyethylene (LDPE). Thus, this study makes contributions on both the open source development of PSO software and finding optimal printing parameters for recycled LDPE.

LDPE has been used to make filament,^54^ but in this study for the first time it is used in the open source FPF/FGF system of the Gigabot X.^55^ The GigabotX is used because it exemplifies a complex 3D printer by printing from pellets, shards, particles, or granules (as opposed to filament), has multiple (3) heat zones in the extruder, and prints on a large scale (570 × 595 × 470 mm).^55^

The optimization is carried out in three steps. First the particles (tests) are defined as 200 mm long singular extrusion lines. Second, the particles are defined as 100 × 100 mm single-layer planes. Third, the particles are defined as 40 × 40 × 40 solid cubes. Each test has pertinent parameters dictated by PSO Experimenter for each particle. To optimize parameters, a fitness function and test set are also proposed. This fitness method is generalizable to all other 3D printing applications.

## Materials and Methods

### Materials and 3D printer

**FIG. 1.**
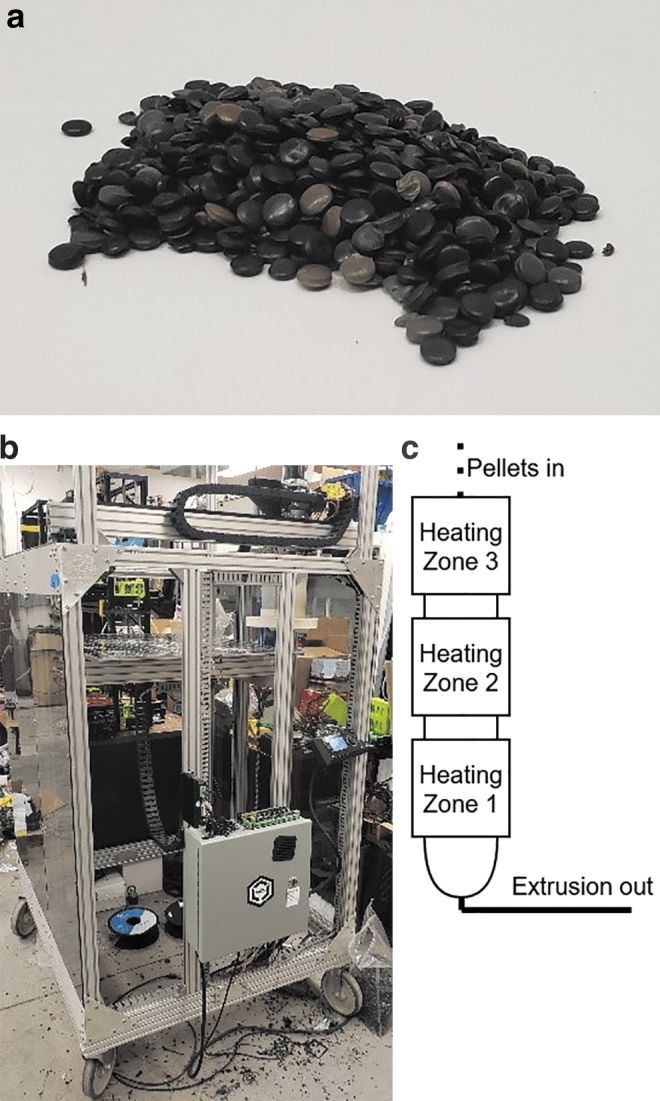
**(a)** Raw LDPE pellets. **(b)** The Gigabot X. **(c)** The three heating zones of the extruder. LDPE, low-density polyethylene.

LDPE pellets (Fig. 1a) were obtained from McDonough Plastics and were 3D printed in a 3-heat-zone Gigabot X (re:3D, Texas) (Fig. 1b). The Gigabot X is a direct pellet material extrusion-based 3D printer with the nozzle arranged vertically in which a compression screw and three hot zones (demonstrated in Fig. 1c) enable a relatively constant flow of recycled material through the print nozzle.

### Software

**FIG. 2.**
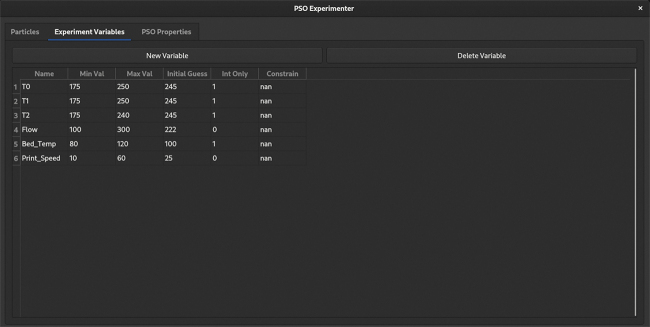
Screen shot of PSO Experimenter during variable entry. PSO, particle swarm optimization.

*PSO Experimenter* is an easy-to-use minimalist optimization platform shown in Figure 2 that utilizes the PSO and is licensed under GPL3.0. PSO consists of a list of particles that have a personal optimum configuration, current position, and a velocity. In the context of PSO, position refers to a certain set of parameters to be experimentally tested. In addition, the group optimum (best of all the personal optimums across all iterations) is known by each particle. PSO is an iterative method, after each iteration the algorithm works to minimize the fitness of each particle.

PSO Experimenter allows users to create an unlimited number of variables, allowing for exploration in *n*-dimensional space. Each variable must have a name, minimum value, maximum value, and an initial guess. In addition, the variable can be constrained to integer values only (input 1 for integer only, 0 for floating point). PSO Experimenter does not allow for addition of new variables after the first iteration is generated, however, variables that may come in to play later in optimization can be constrained by entering a value to constrain at in the constrain column.

Before the first iteration, each particle's position is initialized with a position being a uniformly distributed random vector. PSO Experimenter allows for the input of an initial guess for each variable, and a proximity to that guess. Specifically, the initial position for each particle is generated using Equation (1).^52^

(1) $$x_{i}=U\left(max\left(x_{gi}-\frac{rp}{2},x_{mini}\right),min\left(x_{gi}+\frac{rp}{2},x_{maxi}\right)\right),$$

where *x_i_* is defined as the current position at variable *i*, *U* is the random uniform distribution between two given values, $x_{gi}$ is the guess value for variable *i*, $x_{mini}$ is the minimum allowable value for variable *i*, $x_{maxi}$ is the maximum allowable value for variable *i*, *r* is the range between $x_{mini}$ and $x_{maxi}$, and *p* defines the proximity to $x_{gi}$.

In addition, the particle's velocity is initialized according to Equation (2)^52,56^:

(2) $$v_{i}=U\left(-\left|x_{maxi}-x_{mini}\right|,\left|x_{maxi}-x_{mini}\right|\right),$$

where *v_i_* is the velocity for variable *i*.

For each iteration of the algorithm, the particle's position must be tested and then assigned a resultant fitness. When a new iteration is generated, the particle's velocity is updated by Equation (3)^52,56^: where $x_{gbi}$ is the position in variable *i* for the particle's current personal best and $x_{pbi}$ is the position for the swarm's current group best. The weight parameters are defined in Table 1.

**Table 1.** Main Parameters for Tuning Particle Swarm Optimization Behavior

Variable	Description
*k_v_*	The emphasis given to the velocity component
*k_p_*	The emphasis given to a particle's personal best position
*k_g_*	The emphasis given to the swarm's group best position

(3) $$v_{i}\leftarrow k_{v}v_{i}+k_{p}U\left(0,1\right)\left(x_{pbi}-x_{i}\right)+k_{g}U\left(0,1\right)\left(x_{gbi}-x_{i}\right).$$

Finally, the particles new position at variable *i* is updated in Equation (4)^52,56^:

(4) $$x_{i}\leftarrow x_{i}+v_{i}.$$

The workflow in PSO Experimenter starts with variable entry. The ranges and initial guesses for each variable can be acquired in four different ways:

1. Literature review: Reviewing studies in academia exploring a process similar to the optimization objective can yield information on commonly used parameter values.^24–26^
2. Expert consultation: In cases when the process is completely novel, a subject-matter expert may have intuition on what ranges to search for each given variable.
3. Initial experimentation: Arbitrary, but guided, experimentation can show some parameters that work, and some that do not. These experiments can provide insight into what ranges to search in.
4. Previous optimization experiments^24–26^: The output of a previous PSO experiment can be used as a starting point for further optimizations.

In this study, the first trial's variable parameters are found through literature review on previous GigabotX work^24–26^ and initial experimentation. The two following tests use the values from the previous optimization experiment.

In addition to variable information, the optimization parameters must be entered. These weights can be found in either the literature review, or through experimentation with a test function that has a similar response to the system being optimized. After all variables and parameters are entered, the first iteration can be generated.

**FIG. 3.**
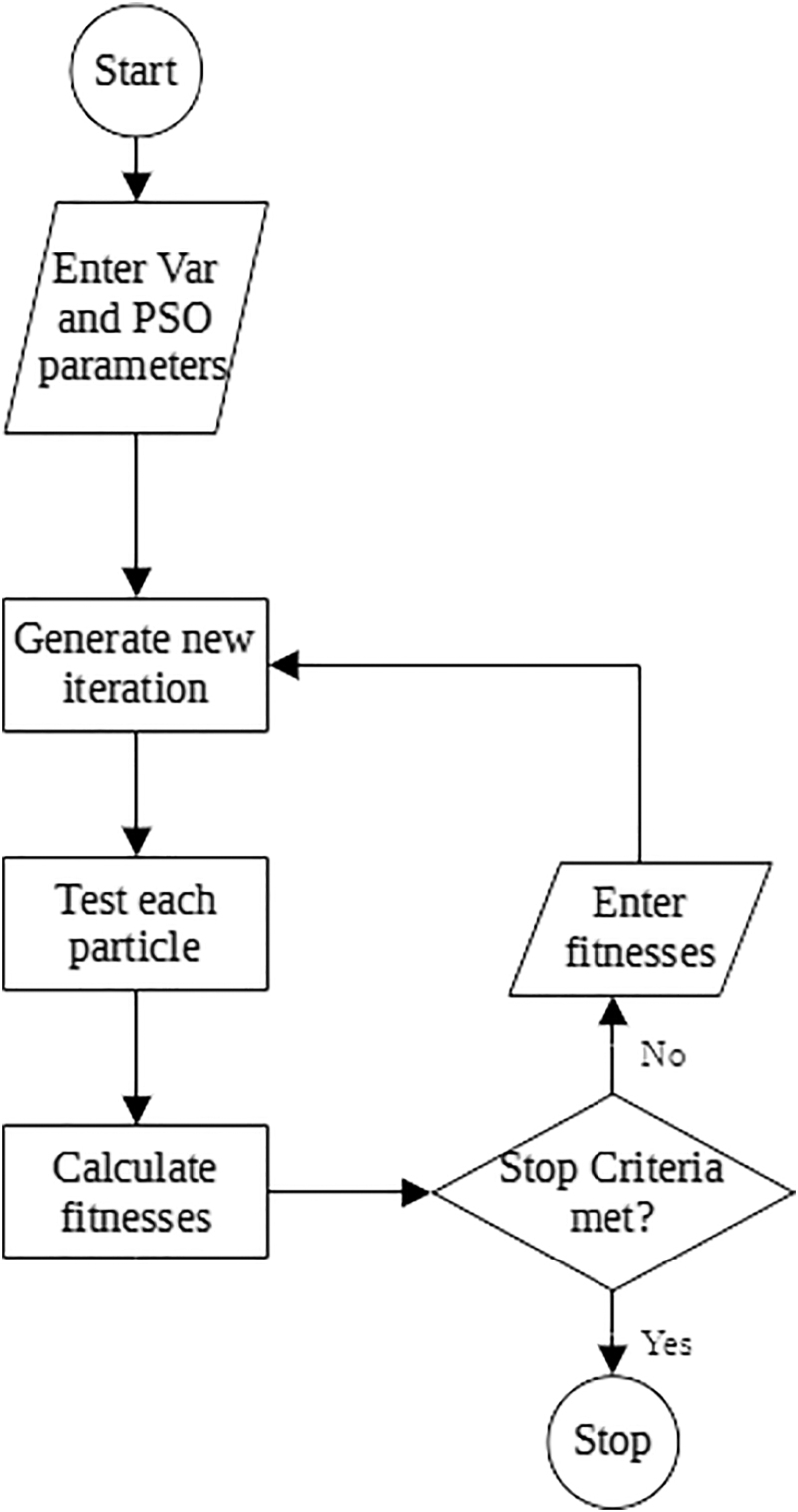
PSO Experimenter general workflow.

The first iteration has a fixed number of particles, each with a unique set of variable values that must be used in the process or experiment. Either during or after (or both) the experiment, measurements must be taken to quantify the success of that particular combination. A fitness function must be established that is a function of the measurements, and trends downward as measurements become more desirable. The simplest fitness function can be a normalized sum of accuracy measurements. The resulting fitness for each particle can be entered into PSO Experimenter, and the next iteration is generated. The optimization process can either be run for a fixed number of iterations, or until the fitness is below a desirable threshold. The general workflow for PSO Experimenter is shown in Figure 3.

PSO Experimenter also allows for saving and loading particle and experiment data. The data are saved in clearly formatted comma-separated values so that historical data can be viewed for further analysis. In addition, this open format allows the user to intervene and change parameters as needed (though this is not always recommended, since errors in alteration can render the file unusable, it can be useful for specialized corrections)

### Parameter acquisition

In this study, the process being optimized is printing recycled waste LDPE pellets using a Gigabot × 3D printer. PSO typically uses thousands of particles in the swarm, but since this experiment requires physical processes and measurements,^57^ only five particles were used to minimize experimental time. Since this number is low, the experimental parameters must be chosen such that the particles are more explorative (rather than exploitive) to prevent early convergence on a local minimum. More explorative particles spend more iterations exploring the sample space, and their velocity slowly decays as they begin to converge.^57^

To find the ideal experimental parameters, PSO Experimenter is used to optimize test functions that have trends similar to the response of a 3D printer. 3D printing is assumed to be a hypervalley—many minima that are very close in fitness to the global minima, stretched out across the sample space.^58^ Functions such as the Beale function [Equation (5)] and the Goldstein–Price function [Equation (6)] are valley like and are used as benchmarks to set expectations for the physical experiments.^59^

(5) $$\begin{array}{c} f\left(x,y\right)={\left(1.5-x+xy\right)}^{2}+{\left(2.25-x+xy^{2}\right)}^{2}\\ +{\left(2.625-x+xy^{3}\right)}^{2}. \end{array}$$

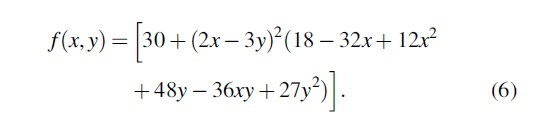


**FIG. 4.**
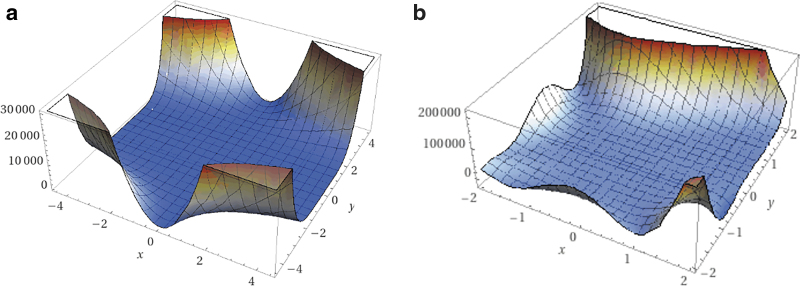
**(a)** Plot of Beale function. **(b)** Plot of Goldstein–Price function.

The Beale function has a minimum of 0 at *f*(3, 0.5), and the Goldstein–Price function has a minimum of 3 at (0,−1). Their respective plots are shown in Figure 4 and represent trends observed in 3D printing material explorations.

Each function is built into a spreadsheet such that the fitness is defined by the *X* and *Y* input values. First PSO Experimenter is configured with a *k_v_* of 0.1, *k_p_* of 1, and *k_g_* of 2. It should be noted that the k values are varied for the algorithm dictates how particles navigate the *X* − *Y* space and are not correlated with the input parameters. The group best is exploitive toward current global minima and personal best favors its personal best. The higher the velocity weight, the more explorative.

Each function is tested for 20 iterations to generate a baseline, and then the velocity weight (which alters how explorative the particles are) is set to 0.5. The responses are compared, and the parameter set that yields the highest accuracy is used in the physical experiments.

### LDPE optimization on Gigabot X experiment design

**FIG. 5.**
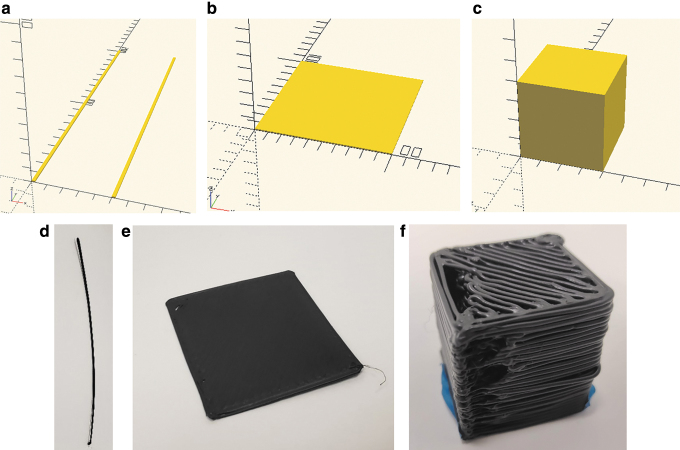
**(a)** A rendering of the line test. **(b)** A rendering of the plane test. **(c)** A rendering of the cube test. **(d)** An image of a resulting line test. **(e)** An image of a resulting plane test. **(f)** An image of a resulting cube test. It should be noted these test examples are before any optimization.

For the first optimization experiment, single 200 mm lines are extruded (Fig. 5a). For this trial, physical dimensions that were measured for the optimization included length (*l*) accuracy, width (*w*) accuracy, width constancy, and mass (*m*) accuracy. Accuracy is defined in Equation (7). Consistency is defined in Equation (8), where the measurements are evenly distributed along the length of the extrusion. The initial variable parameters are listed in Table 2.

**Table 2.** Experimental Input Parameters for Line Optimization

Variable	Min	Max	Guess	True/False	Description
*T*_1_	175°C	250°C	245°C	True	Temperature zone 1 on Gigabot X extruder
*T*_2_	175°C	250°C	245°C	True	Temperature zone 2 on Gigabot X extruder
*T*_3_	175°C	250°C	245°C	True	Temperature zone 3 on Gigabot X extruder
*F*	100%	300%	222%	False	Extrusion flow multiplier
*T_B_*	80°C	120°C	100°C	True	Bed temperature
*V_P_*	10 mm/s	60 mm/s	25 mm/s	False	Print speed (end effector movement rate)

(7) $$A\left(X_{m},X_{d}\right)=\frac{\left|X_{m}-X_{d}\right|}{X_{d}},$$

where *X_m_* is the measured value (or in some cases the averaged measured value), and *X_d_* is the desired value.

(8) $$C\left(X_{m1},X_{m2}\ldots X_{mn}\right)=avgdev\left(X_{m1},X_{m2},\ldots ,X_{mn}\right).$$

The fitness function is defined by the weighted sum of the measurements [Equation (9)]. The weights are assigned according to expert knowledge. The experiment uses optimization parameters found from the test functions in the previous step, and is run for either 20 iterations, or until the fitness is <0.1.

(9) $$\begin{array}{c} f_{line}=0.2C\left(w_{m1},\ldots ,w_{m5}\right)+0.2A\left(w_{avg},2.2mm\right)\\ +0.1A\left(l_{m},200mm\right)+0.5A\left(m_{m},0.4g\right), \end{array}$$

where the physical dimensions are length (*l*) accuracy, width (*w*) accuracy, width constancy, and mass (*m*) accuracy, where the subscript m is measured and the average is already described. The second trial is optimizing the length accuracy, width accuracy, height consistency, height accuracy, and mass accuracy of 100 by 100 mm planes (Fig. 5b). The optimization input parameters remains the same with the addition of *E*, the edge overlap of the infill percentage (with a range of 10–20%, and initial guess of 15%). The initial guess values are selected as the optimum parameters from the previous trial (with a proximity value of 0.25). For this trial, additional factors including layer height (*h*) and print time (*t*) are also considered. The fitness function is defined in Equation (10).

(10) $$\begin{array}{c} f_{plane}=0.1C\left(h_{m1},\ldots ,w_{m5}\right)+0.1A\left(h_{avg},1.01mm\right)\\ +0.1A\left(l_{avg},100mm\right)+0.1A\left(w_{avg},100mm\right)\\ +0.1A\left(t_{m},6.5min\right)+0.5A\left(m_{m},9.4g\right) \end{array}$$

The final trail optimizes the height, width, length, and mass accuracy of a 40 by 40 by 40 mm cube (Fig. 5c). The optimization parameters once again remain the same with the inclusion of the layer height *H_L_* ranging from 0.6 to 1.5 mm, and infill density *D_I_* ranging from 100% to 250%. The initial guess is selected as the optimum parameters from the plane trial (with a proximity of 0.25). The fitness function for this trial is given in Equation (11).

(11) $$\begin{array}{c} f_{cube}=0.1A\left(w_{avg},40mm\right)+0.1A\left(l_{avg},40mm\right)\\ +0.1A\left(h_{avg},40mm\right)+0.7A\left(m_{m},58.2g\right). \end{array}$$

## Results and Discussion

### Parameter acquisition

**FIG. 6.**
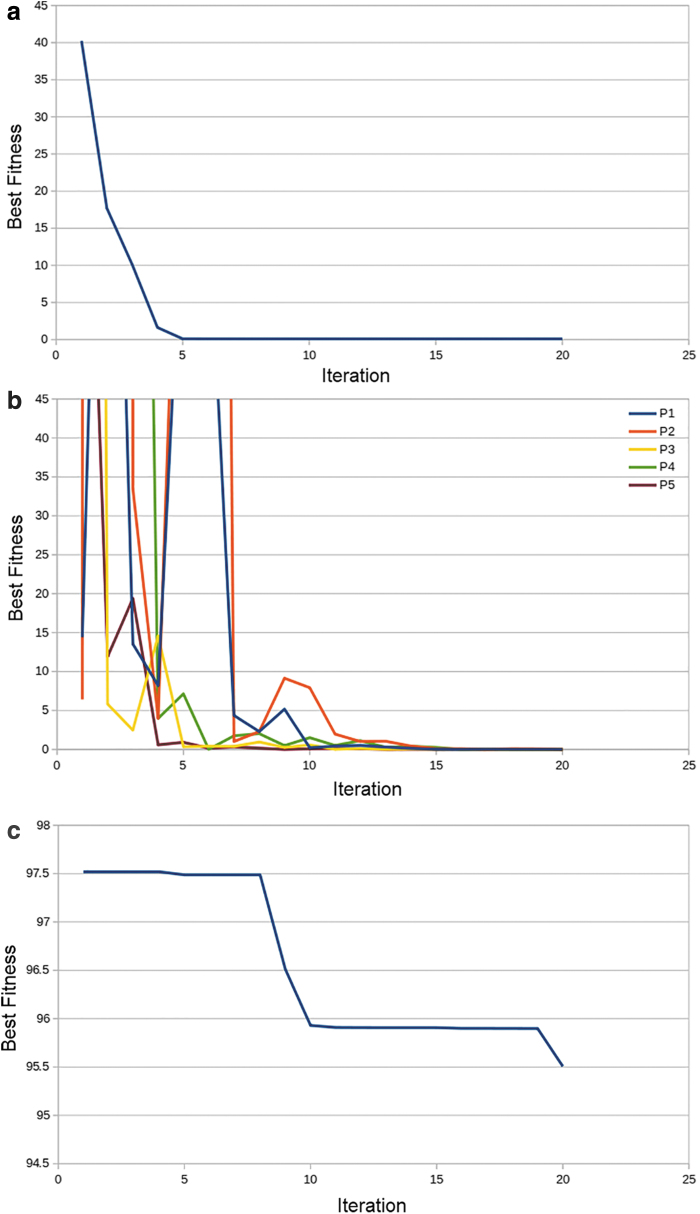

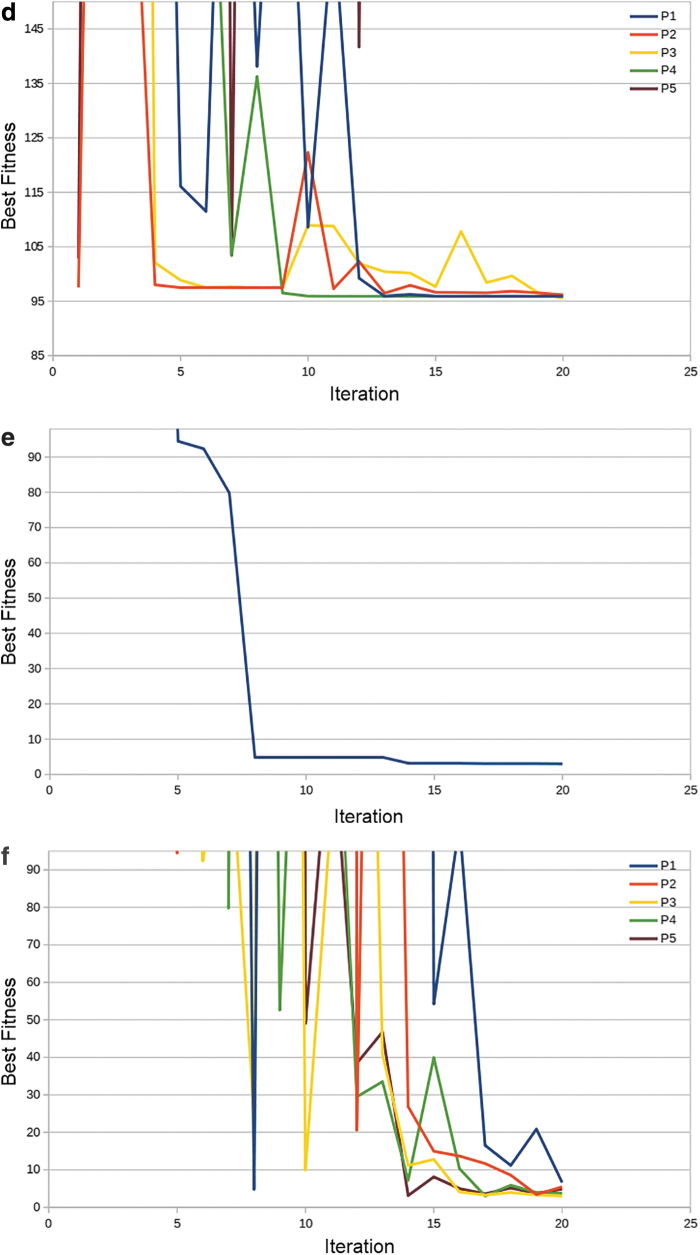
Performance of PSO on Beale function: **(a)** Total best fitness. **(b)** Particle fitness over time performance of PSO on Goldstein–Price function. **(c)** Total best fitness with *k_v_* = 0.1. (**d)**. Particle fitness over time *k_v_* = 0.1. **(e)** Total best fitness with *k_v_* = 0.5. **(f)** Particle fitness over time *k_v_* = 0.5.

Twenty iterations of optimization on the Beale function were run on the waste LDPE on the Gigabot X. The individual experiment fitnesses (particles) and group best fitness are shown in Figure 6. The Best Fitness on the left (Fig. 6 a, c, e) is the fitness of the group best, where Figure 6b, d, and f is (right ones) the fitness of the personal bests.

Next, 20 iterations of optimization on the Golstein–Price function were run on the same material and process system. The individual particle fitnesses and group best fitness are shown in Figure 6. Then optimization is run with a velocity weight of 0.5. The group fitnesses between both trials can be compared. The hypothesis that a higher velocity weight will cause more exploration has been confirmed, and in addition enabled particles to converge on a more desirable fitness value. This indicates that the parameters listed in Table 3 should be used for 3D print optimization.

**Table 3.** Recommended Parameters for Particle Swarm Optimization Tuning of 3D Printing

Variable	Value
*k_v_*	0.5
*k_p_*	1
*k_g_*	2

In addition, from Figure 6a, the physical experiment should expect to see a convergence around six iterations. This experiment set successfully demonstrated that PSO Experimenter should be attempted for 3D printer optimization.

### LDPE optimization on Gigabot X results

**FIG. 7.**
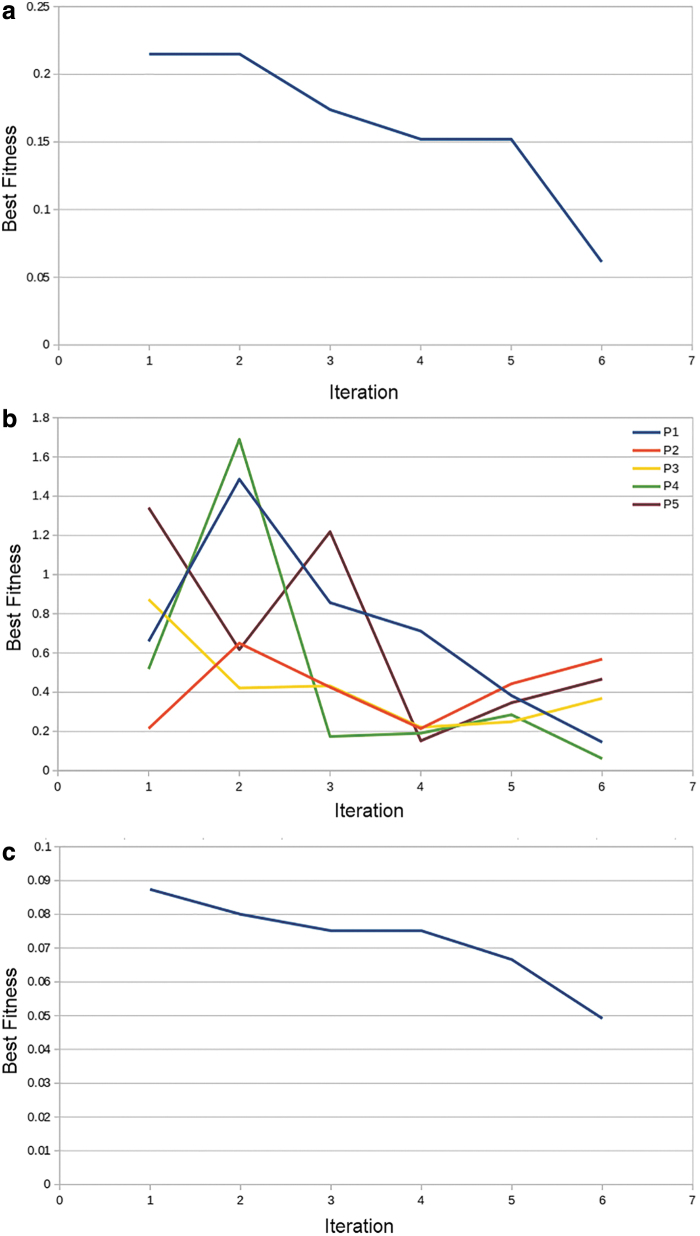

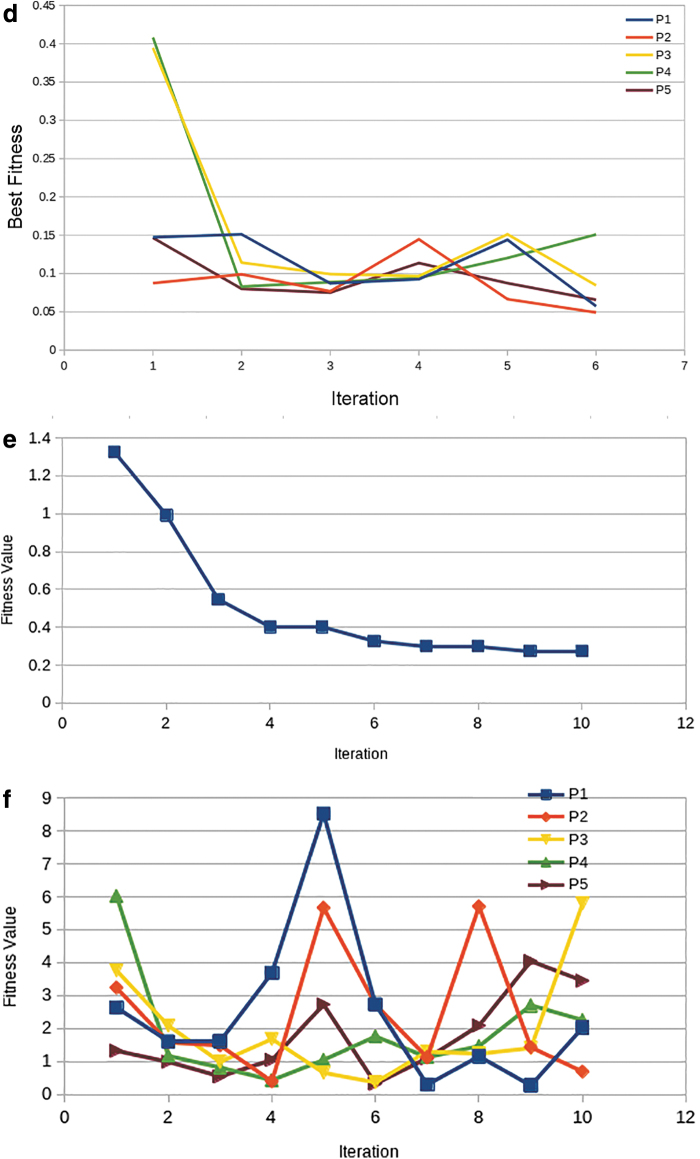
Performance of PSO on line tests: **(a)** Total best fitness. **(b)** Particle fitness over time. Performance of PSO on plane tests: **(c)** Total best fitness. **(d)** Particle fitness over time. Performance of PSO on cube tests: **(e)** Total best fitness and **(f)** particle fitness over time.

The line trial was able to reach a fitness of <0.1 after six iterations. The optimization performance is shown in Figure 7. The ideal parameters for printing lines are listed in column 3 of Table 4.

**Table 4.** Ideal Printing Parameters for Fused Particle Fabrication/Fused Granule Fabrication of Waste Low-Density Polyethylene on a Three Temperature Zone Extruder

Variable	Description	Lines value	Planes value	Cube value
*T*_1_	Temperature zone 1 on Gigabot X extruder	225°C	220°C	225°C
*T*_2_	Temperature zone 2 on Gigabot X extruder	200°C	200°C	205°C
*T*_3_	Temperature zone 3 on Gigabot X extruder	175°C	172°C	166°C
*F*	Extrusion flow multiplier	150%	165%	156%
*T_B_*	Bed temperature	90°C	95°C	94°C
*V_P_*	Print speed (end effector movement rate)	10 mm/s	38 mm/s	35 mm/s
*E*	Edge overlap of infill	NA	18%	18%
*H_L_*	Layer height	NA	NA	0.6mm
*D_I_*	Infill density	NA	NA	165%

**FIG. 8.**
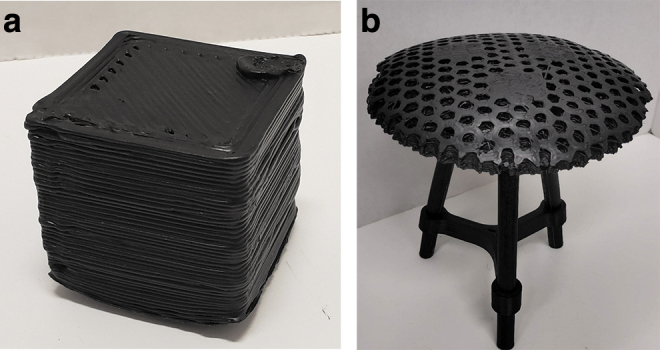
**(a)** Optimized print setting results of LDPE waste printed into a test cube and **(b)** an LDPE 3D printed stool base on PETG legs. PETG, glycol modified polyethylene terephthalate.

The plane trial was able to reach a fitness of <0.1 after one iteration. The optimization performance is shown in Figure 8. The ideal parameters for printing planes are listed in column 4 of Table 4.

The cube trial was not able to reach a fitness of 0.1 after six iterations. Due to machine limitations (inability to make perfectly square corners causes the volume estimate to be imprecise) the goal fitness may not be achievable. The optimization performance is shown in Figure 7. The ideal parameters for printing lines are listed in column 5 of Table 4, and these parameters are accepted as ideal parameters for general use in printing with LDPE. An additional result of this experimentation is the finding that the print surface should be covered in clear PP packaging tape for optimum adhesion.

The optimization improved the print quality as shown in Figure 7, an optimized test cube, which can be compared directly with Figure 5f. The print is not a complete digital replication of the design as there still is some deformation at the base and not quite perfect final layer print. This is somewhat expected because LDPE is a known challenging 3D printing material with only a few vendors offering it.^60^

The printing quality, however, is high enough to be used for large (human)-scale functional objects as this stool is as shown in Figure 8b. The device has a mass of 0.56 lb, which at a cost of $1/lb and 0.28 lb of glycol modified polyethylene terephthalate (PETG) used for the legs at $9.50 per pound^61^ results in a cost for the stool of $3.22. This is an 87% savings from commercial devices that can cost >$24,^62^ and could be further reduced using reclaimed materials for legs. These results agree with past work,^63^ which found substantial economic potential for DRAM,^61^ including specific investigations on the potential for large-scale 3D printing of athletic equipment from plastic waste.^49^

### Impact and applications

The goal of this study was to reduce the time to obtain optimal 3D printing parameters from waste plastic feedstocks. The new methodology and open source software were able to accomplish this goal. The time for preparation, printing, and measurement of a line sample takes ∼10 min. With the first physical trial settling below a fitness of 0.1 after six iterations, the total time elapsed is 5 h. If this same experiment were to be run as a conservative matrix test, (where temperature zones are all kept at the same temperature and tested across 10 increments, bed temperature is kept at a constant, and feed rate and print speed's range was cut into 10 increments) it would require over 166 h.

This indicates that PSO experiment can provide 97% reduction in research time for 3D printing optimization. Because of this, PSO experiment is recommended for all future material parameter acquisition, as it can explore the sample space with self-adjusting granularity and can substantially reduce time, money, and material usage. This is in line with past work that showed PSO utility for build orientation^64^ support structures^65^ and fused filament 3D printing.^66^

This accelerated testing for new materials is particularly useful in the DRAM context as the 3D printing process parameter optimization may need to be completed for each new waste material source, even if the primary polymer is the same. This is because past results have shown that not only do additives and processing matter but so do color,^30^ number of cycles,^9^ water absorption, and thus storage and thermal history as well as source^24^ can impact 3D printing parameter optimization and final properties of the manufactured part.

PSO Experimenter could be used to optimize not only waste materials but also various other materials such as PP or HDPE, which are not yet commonly used in commercial fused filament-based 3D printing because of their challenging thermal expansion coefficients. In addition, the software could be used to take the guess work out of tuning and calibrating machines such as circuit mills,^67^ metal 3D printers,^68^ and bioprinters.^1^

### PSO versus random selection

PSO is not a random walk,^52^ it has a directed search that lowers optimization time. As a comparison, if the optimization process was defined such that there are 1000 combinations of variables, the probability of randomly selecting the optimum is 0.1%. Considering an equal number of trials to the PSO (30 per experiment) as Bernoulli trials with a success probability of 0.1%, the probability of successfully identifying a global optimum can be modeled as a binomial random variable.^69^ This model shows that arbitrary guessing of combinations yields a 2.91% chance of successfully arriving at the optimum.

Clearly each optimization run performed in this article has arrived at acceptable optimums, indicating that the PSO algorithm is aiding in the process and it is not pure randomness. This is in agreement with past research that has applied PSO to 3D printing parameter optimization such as Shirke *et al.*'s analysis of maximizing tensile strength^70^ and Shen *et al.*'s analysis of build orientation.^64^

### Future study

PSO Experimenter can be further improved by being integrated directly into a printer control software,^71^ to directly control the machine for experiments. In addition, PSO Experimenter can be applied to a myriad of diverse applications such as custom filament extrusion, circuit board milling, recipes, and farming. To improve the success of LDPE recycling, methods to gain a better understanding of the composition of the postconsumer plastics are necessary.^72–74^ It should be pointed out that the material properties or recycled materials and composites may impact the appropriateness of 3D printed parts for some applications.

Thus, even if the PSO Experimenter provides optimal printing parameters, the recycled material may not have adequate properties (e.g., tensile strength is reduced with each recycling cycle) and the thermomechanical environment in the extrusion process also can impact the strength even if the physical dimensions are correct. Future study is needed to look carefully at the degradation effect of DRAM. Future study is also needed to improve slicing and other means to overcome the warping observed with large LDPE prints. Lastly, there has been progress on development of custom screws for DRAM,^75^ and information from Justino *et al.*,^76^ which reported a systematic review of screw-assisted 3D printing equipment, could be used for optimizing additive manufacturing (AM) of LDPE with screw extrusion technology.

## Conclusions

PSO Experimenter was created to expedite the acquisition of ideal process parameters. As a case study, the free and open software was used to find ideal parameters for recycled waste LDPE direct extrusion 3D printing using an open source AM system. The results showed that the algorithm was able to find ideal parameters in six iterations, taking a time of 5 h. This is a substantially less amount of time to get functional printing parameters. Overall, there is a 97% reduction in time used compared to matrix-based process parameter testing.

It can be concluded that the PSO Experimenter was able to find the optimum parameters setting for recycle LDPE materials. Overall the results of the study demonstrated that the PSO Experimenter is a user-friendly and effective free software for finding ideal parameters for DRAM and it could be used as well in a wide range of other fields and processes.

## References

[1] Krige A, Haluška J, Rova U, *et al.* Design and implementation of a Low Cost Bio-Printer Modification, allowing for switching between plastic and gel extrusion. HardwareX 2021;9. DOI: 10.1016/j.ohx.2021.e00186 PMC9041258 35492054

[2] Abdollahi S, Davis A, Miller JH, *et al.* Expert-guided optimization for 3D printing of soft and liquid materials. PLoS ONE 2018;13:e0194890. 29621286 10.1371/journal.pone.0194890 PMC5886457

[3] Lee J-Y, An J, Chua CK. Fundamentals and applications of 3D printing for novel materials. Appl Mater Today 2017;7:120–133.

[4] Kulkarni A, Sorarù GD, Pearce JM. Polymer-derived SiOC Replica of material extrusion-based 3-D printed plastics. Addit Manuf 2020;32:100988.

[5] Veteška P, Hajdúchová Z, Feranc J, *et al.* Novel composite filament usable in low-cost 3D printers for fabrication of complex ceramic shapes. Appl Mater Today 2021;22:100949.

[6] Menon A, Póczos B, Feinberg AW, *et al.* Optimization of silicone 3D Printing with hierarchical machine learning. 3D Print Addit Manuf 2019;6:181–189.

[7] Feng C, Zhang M, Bhandari B. Materials properties of printable edible inks and printing parameters optimization during 3D printing: A review. Crit Rev Food Sci Nutr 2019;59:3074–3081. 29856675 10.1080/10408398.2018.1481823

[8] Hunt EJ, Zhang C, Anzalone N, *et al.* Polymer recycling codes for distributed manufacturing with 3-D printers. Resour Conserv Recycl 2015;97:24–30.

[9] Sanchez FAC, Lanza S, Boudaoud H, *et al.* Polymer Recycling and Additive Manufacturing in an Open Source Context: Optimization of Processes and Methods; Annual International Solid Freeform Fabrication Symposium, ISSF 2015, Aug 2015, Austin, TX, United States. pp. 1591–1600.

[10] Cruz Sanchez FA, Boudaoud H, Hoppe S, *et al.* Polymer recycling in an open-source additive manufacturing context: Mechanical issues. Addit Manuf 2017;17:87–105.

[11] Anderson I. Mechanical properties of specimens 3D printed with virgin and recycled polylactic acid. 3D Print Addit Manuf 2017;4:110–115.

[12] Pakkanen J, Manfredi D, Minetola P, *et al.* About the use of recycled or biodegradable filaments for sustainability of 3D Printing. In: Sustainable Design and Manufacturing 2017. (Campana G, Howlett RJ, Setchi R, *et al.* eds). Cham: Smart Innovation, Systems and Technologies Springer International Publishing, 2017; pp.776–785.

[13] Zhong S, Pearce JM. Tightening the loop on the circular economy: Coupled distributed recycling and manufacturing with Recyclebot and RepRap 3-D Printing. Resour Conserv Recycl 2018;128:48–58.

[14] Mohammed MI, Wilson D, Gomez-Kervin E, *et al.* Investigation of closed-loop manufacturing with acrylonitrile butadiene styrene over multiple generations using additive manufacturing. ACS Sustain Chem Eng 2019;7:13955–13969.

[15] Mohammed MI, Wilson D, Gomez-Kervin E, *et al.* The Recycling of E-Waste ABS Plastics by Melt Extrusion and 3D Printing Using Solar Powered Devices as a Transformative Tool for Humanitarian Aid. University of Texas at Austin, 2018.

[16] Mohammed MI, Wilson D, Gomez-Kervin E, *et al.* EcoPrinting: Investigation of solar powered plastic recycling and additive manufacturing for enhanced waste management and sustainable manufacturing. In: 2018 IEEE Conference on Technologies for Sustainability (SusTech), 2018; pp.1–6. DOI:10.1109/SusTech.2018.8671370

[17] Boldizar A, Möller K. Degradation of ABS during repeated processing and accelerated ageing. Polym Degrad Stab 2003;81:359–366.

[18] Baechler C, DeVuono M, Pearce JM. Distributed recycling of waste polymer into RepRap Feedstock. Rapid Prototyp J 2013;19:118–125.

[19] Chong S, Pan G-T, Khalid M, *et al.* Physical characterization and pre-assessment of recycled high-density polyethylene as 3D Printing Material. J Polym Environ 2017;25:136–145.

[20] Pepi M, Zander N, Gillan M. Towards expeditionary battlefield manufacturing using recycled, reclaimed, and scrap materials. JOM 2018;70:2359–2364.

[21] Oblak P, Gonzalez-Gutierrez J, Zupančič B, *et al.* Processability and mechanical properties of extensively recycled high density polyethylene. Polym Degrad Stab 2015;114:133–145.

[22] Woern A, Pearce J. Distributed manufacturing of flexible products: technical feasibility and economic viability. Technologies 2017;5:71.

[23] Lee JH, Lim KS, Hahm WG, *et al.* Properties of recycled and virgin poly(ethylene terephthalate) blend fibers. J Appl Polym Sci 2013;128:1250–1256.

[24] Little HA, Tanikella NG, J. Reich M, *et al*. Towards distributed recycling with additive manufacturing of PET Flake Feedstocks. Materials 2020;13:4273. 32992735 10.3390/ma13194273 PMC7578976

[25] Reich MJ, Woern AL, Tanikella NG, *et al.* Mechanical properties and applications of recycled polycarbonate particle material extrusion-based additive manufacturing. Materials 2019;12:1642. 31137505 10.3390/ma12101642 PMC6566670

[26] Woern AL, McCaslin JR, Pringle AM, *et al.* RepRapable Recyclebot: Open source 3-D printable extruder for converting plastic to 3-D printing filament. HardwareX 2018;4:e00026.

[27] Pavlo S, Fabio C, Hakim B, *et al.* 3D-printing based distributed plastic recycling: A conceptual model for closed-loop supply chain design. In: 2018 IEEE International Conference on Engineering, Technology and Innovation (ICE/ITMC), 2018; pp.1–8. DOI: 10.1109/ICE.2018.8436296

[28] Cruz Sanchez FA, Boudaoud H, Camargo M, *et al.* Plastic recycling in additive manufacturing: A systematic literature review and opportunities for the circular economy. J Clean Prod 2020;264:121602.

[29] Gwamuri J, Wittbrodt BT, Anzalone NC, *et al.* Reversing the trend of large scale and centralization in manufacturing: The case of distributed manufacturing of customizable 3-D-printable self-adjustable glasses. Chall Sustain 2014;2:30–40.

[30] Wittbrodt BT, Glover AG, Laureto J, *et al.* Life-cycle economic analysis of distributed manufacturing with Open-Source 3-D Printers. Mechatronics 2013;23:713–726.

[31] Petersen E, Pearce J. Emergence of home manufacturing in the developed world: Return on investment for Open-Source 3-D Printers. Technologies 2017;5:7.

[32] Petersen E, Kidd R, Pearce J. Impact of DIY home manufacturing with 3D Printing on the Toy and Game Market. Technologies 2017;5:45.

[33] Laplume AO, Petersen B, Pearce JM. Global value chains from a 3D printing perspective. J Int Bus Stud 2016;47:595–609.

[34] Kreiger M, Anzalone GC, Mulder ML, *et al.* Distributed recycling of post-consumer plastic waste in rural areas. MRS Proc 2013;1492:91–96.

[35] Kreiger MA, Mulder ML, Glover AG, *et al.* Life cycle analysis of distributed recycling of post-consumer high density polyethylene for 3-D printing filament. J Clean Prod 2014;70:90–96.

[36] Zhong S, Rakhe P, Pearce J. Energy payback time of a solar photovoltaic powered waste plastic recyclebot system. Recycling 2017;2:10.

[37] Tian X, Liu T, Wang Q, *et al.* Recycling and remanufacturing of 3D printed continuous carbon fiber reinforced PLA composites. J Clean Prod 2017;142:1609–1618.

[38] Parandoush P, Lin D. A review on additive manufacturing of polymer-fiber composites. Compos Struct 2017;182:36–53.

[39] Heller BP, Smith DE, Jack DA. Planar deposition flow modeling of fiber filled composites in large area additive manufacturing. Addit Manuf 2019;25:227–238.

[40] Pringle AM, Rudnicki M, Pearce JM. Wood furniture waste–based recycled 3-D printing filament. Forest Prod J 2018;68:86–95.

[41] Zander NE. Recycled polymer feedstocks for material extrusion additive manufacturing. In: Polymer-Based Additive Manufacturing: Recent Developments. ACS Symposium Series 1315 American Chemical Society, 2019; pp.37–51. DOI:10.1021/bk-2019-1315.ch003

[42] Dertinger SC, Gallup N, Tanikella NG, *et al.* Technical pathways for distributed recycling of polymer composites for distributed manufacturing: Windshield Wiper Blades. Resour Conserv Recycl 2020;157:104810.

[43] Meyer TK, Tanikella NG, Reich MJ, *et al.* Potential of distributed recycling from hybrid manufacturing of 3-D printing and injection molding of stamp sand and acrylonitrile styrene acrylate waste composite. Sustain Mater Technol 2020;25:e00169.

[44] Zander NE, Gillan M, Burckhard Z, *et al.* Recycled polypropylene blends as Novel 3D Printing Materials. Addit Manuf 2019;25:122–130.

[45] Pearce J. Expanding the consumer bill of rights for material ingredients. Mater Today 2018;21:197–198.

[46] Shakor P, Nejadi S, Sutjipto S, *et al.* Effects of deposition velocity in the presence/absence of E6-Glass Fibre on Extrusion-Based 3D Printed Mortar. Addit Manuf 2020;32:101069.

[47] Woern A, Byard D, Oakley R, *et al.* Fused particle fabrication 3-D printing: Recycled materials’ optimization and mechanical properties. Materials 2018;11:1413. 30103532 10.3390/ma11081413 PMC6120030

[48] Alexandre A, Cruz Sanchez FA, Boudaoud H, *et al.* Mechanical properties of direct waste printing of polylactic acid with Universal Pellets Extruder: Comparison to fused filament fabrication on Open-Source Desktop Three-Dimensional Printers. 3D Print Addit Manuf 2020;7:237–247.

[49] Byard DJ, Woern AL, Oakley RB, *et al.* Green Fab Lab Applications of large-area waste polymer-based additive manufacturing. Addit Manuf 2019;27:515–525.

[50] Nilsiam Y, Sanders P, Pearce JM. Slicer and process improvements for open-source GMAW-Based Metal 3-D Printing. Addit Manuf 2017;18:110–120.

[51] Keller JM, Liu D, Fogel DB. Fundamentals of Computational Intelligence: Neural Networks, Fuzzy Systems, and Evolutionary Computation. Hoboken, New Jersey, United States, John Wiley & Sons, 2016.

[52] Saad MS, Nor AM, Baharudin ME, *et al.* Optimization of surface roughness in fdm 3d printer using response surface methodology, particle swarm optimization, and symbiotic organism search algorithms. Int J Adv Manuf Technol 2019;105:5121–5137.

[53] Oberloier S. PSO Experimenter 2021. https://osf.io/muevs/ (accessed July 22, 2021).

[54] Hart KR, Frketic JB, Brown JR. Recycling Meal-Ready-to-Eat (MRE) pouches into polymer filament for material extrusion additive manufacturing. Addit Manuf 2018;21:536–543.

[55] Re3D. Gigabot X Update—Re:3D | Life-Sized Affordable 3D Printing. https://re3d.org/gigabotx-update/ (accessed July 7, 2021).

[56] Banks A, Vincent J, Anyakoha C. A review of particle swarm optimization. Part I: Background and development. Nat Comput 2007;6:467–484.

[57] Wang D, Tan D, Liu L. Particle swarm optimization algorithm: An overview. Soft Comput 2018;22:387–408.

[58] Tanikella NG, Wittbrodt B, Pearce JM. Tensile strength of commercial polymer materials for fused filament fabrication 3D printing. Addit Manuf 2017;15:40–47.

[59] Bossek J. Smoof: Single- and multi-objective optimization test functions. R J 2017;9:103.

[60] Filaments.ca. LLDPE Filament—Natural—1.75mm—1KG, Filaments.Ca. 2021. https://filaments.ca/products/lldpe-filament-natural-1-75mm (accessed October 26, 2021).

[61] Amazon.com: Amazon Basics PETG 3D Printer Filament, 1.75mm, Black, 1 kg Spool: Industrial & Scientific. 2021. https://www.amazon.com//dp/B07T4X4D2C/ (accessed November 13, 2021).

[62] Amazon.com: DOITOOL Plastic Stool Childrens Stool Household Stool Anti- Slip Seat Stool Chair for Living Room Study Room Bathroom: Home & Kitchen. https://www.amazon.com/dp/B091GMYGTR/ (accessed October 25, 2021).

[63] Santander P, Cruz Sanchez FA, Boudaoud H, *et al.* Closed loop supply chain network for local and distributed plastic recycling for 3D printing: A MILP-based optimization approach. Resour Conserv Recycl 2020;154:104531.

[64] Shen H, Ye X, Xu G, *et al.* 3D Printing build orientation optimization for Flexible Support Platform. RPJ 2020;26:59–72.

[65] Zhu L, Feng R, Li X, *et al.* A tree-shaped support structure for additive manufacturing generated by using a hybrid of Particle Swarm Optimization and Greedy Algorithm. J Comput Inf Sci Eng 2019;19:041010.

[66] Dey A, Hoffman D, Yodo N. Optimizing multiple process parameters in fused deposition modeling with particle swarm optimization. Int J Interact Des Manuf 2020;14:393–405.

[67] Oberloier S, Pearce J. Belt-driven open source circuit mill using low-cost 3-D printer components. Inventions 2018;3:64.

[68] Nilsiam Y, Sanders PG, Pearce JM. Applications of open source GMAW-based metal 3-D printing. J Manuf Mater Process 2018;2:18.

[69] Leon-Garcia A. Probability, Statistics, and Random Processes for Electrical Engineering. Hoboken, New Jersey, United States, Prentice Hall, 2008.

[70] Shirke A, Choudhari C, Rukhande S. Parametric optimization of fused deposition modelling (FDM) process using PSO Algorithm. In International Conference on Advances in Thermal Systems, Materials and Design Engineering (ATSMDE2017), Elsevier, Netherlands. DOI: 10.2139/ssrn.3101978

[71] Wijnen B, Anzalone GC, Haselhuhn AS, *et al.* Free and open-source control software for 3-D Motion and Processing. J Open Res Softw 2016;4:e2.

[72] Su Q-Z, Vera P, Salafranca J, *et al.* Decontamination efficiencies of post-consumer high-density polyethylene milk bottles and prioritization of high concern volatile migrants. Resour Conserv Recycl 2021;171:105640.

[73] Cabanes A, Strangl M, Ortner E, *et al.* Odorant composition of post-consumer LDPE bags originating from different collection systems. Waste Manage 2020;104:228–238. 10.1016/j.wasman.2020.01.021 31982786

[74] Demets R, Roosen M, Vandermeersch L, *et al.* Development and application of an analytical method to quantify odour removal in plastic waste recycling processes. Resour Conserv Recycl 2020;161:104907.

[75] Franz J, Pearce JM. Open-source grinding machine for compression screw manufacturing. Inventions 2020;5:26.

[76] Justino Netto JM, Idogava HT, Frezzatto Santos LE, *et al.* Screw-assisted 3D printing with granulated materials: A systematic review. Int J Adv Manuf Technol 2021;115:2711–2727. 34092883 10.1007/s00170-021-07365-z PMC8169388

